# Prevalence of Age-Associated Testosterone Deficiency Syndrome in Indian Population

**DOI:** 10.1155/2019/2468926

**Published:** 2019-03-20

**Authors:** Ghanendra Kumar Yadav, Mrinal Pahwa, Mahendra Singh, Vipin Tyagi, Sudhir Chadha

**Affiliations:** Department of Urology, Sir Ganga Ram Hospital, New Delhi, India

## Abstract

Testosterone deficiency syndrome (TDS) is a gradual age-related phenomenon that occurs in a large proportion of the aging male population. This current prospective study was done with the objective to estimate the prevalence of age-associated TDS in India and its clinical profile. A total of 800 male patients aged ≥40 year were approached to participate in the study. A brief history and focused examination was done. Based on our exclusion criteria, 55 patients were excluded. Androgen deficiency in aging male (ADAM) questionnaire was administered to all remaining 745 patients. Out of these 745 patients, ADAM-positive (symptomatic TDS) patients were found to be 359 and enrolled in the study. In all ADAM-positive patients, serum testosterone levels were measured. Prevalence of symptomatic TDS in study population was found to be 48.18%. Mean total and free testosterone level of symptomatic TDS population were 3.287 ± 1.494 ng/ml (1.12–9.61) and 7.476 ± 2.902 pg/ml (2.18–21.76), respectively. Prevalence of biochemically confirmed TDS among symptomatic TDS population was 60.17%. Prevalence of TDS increases progressively with each decade of life (*p*=0.034). Prevalence was higher in patients with diabetes, hypertension, coronary artery disease, obesity, and metabolic syndrome. TDS is a real phenomenon with a prevalence of 28.99% in our study population.

## 1. Introduction

Normal aging frequently results in a progressive functional dwindling of hypothalamic-pituitary-gonadal axis. This results in progressive decline in serum testosterone in men after age of forty between 0.4 and 2.6% per year [1–4], which exhibits significant interindividual variability in age of onset, speed, and depth of decline [1]. This decline of the serum testosterone level is clinically termed as partial androgen deficiency of the aging male (PADAM) or late-onset hypogonadism (LOH) or more accurately testosterone deficiency syndrome (TDS), but the time of onset varies and the epidemiological status is unclear [5].

TDS is defined as a clinical and biochemical syndrome associated with advancing age and characterized by symptoms with or without signs and a deficiency in serum testosterone levels. This condition may result in significant detriment in the quality of life and adversely affect the function of multiple organ systems [1]. The presence of symptoms, however, is a sine qua non for diagnosis and should be coupled with a low or borderline-low objective biochemical measure of serum testosterone [5–8].

The main emphasis of healthcare in the 21st century is to improve the quality of life of the elderly. In women, hormone replacement therapy is a widely accepted concept. Age-associated TDS and the use of exogenous testosterone to treat decreasing testosterone levels in older men is a relatively new concept [6], and most literature is from European or American continents [7, 8].

In Indian subcontinent, this syndrome has been considered as a general phenomenon associated with aging and very few studies have been carried out to document its true prevalence and clinical profile, resulting in a lack of objective treatment for elderly men [9, 10].

## 2. Materials and Methods

This prospective study was conducted in accordance with the hospital ethical committee requirements in the Department of Urology at a tertiary hospital in New Delhi. A total of 800 male patients aged ≥40 year admitted or attending outpatient department in our department were approached to participate in the study. They were given written information about the study, and informed consent was obtained. A brief history and focused examination was done. Patients with a history of suboptimal pubertal development, medical or surgical castration, dyslipidemia, and chronic liver disease, undergoing androgen supplement therapy, and using drugs which interfere with testosterone levels were excluded. Based on our exclusion criteria, 55 patients were excluded.

ADAM questionnaire was administered to all remaining 745 patients. Symptomatic TDS was considered to be present if at least question numbers 1 or 7 or any 3 other questions of the questionnaire were positive (Table 1).

Out of these 745 patients, 359 patients were found to be suffering from symptomatic TDS based on the ADAM questionnaire and enrolled in the study. In all these ADAM-positive patients, serum total testosterone (TT) and serum free testosterone (FT) levels were measured by the radioimmunoassay method between 07 : 00 and 10 : 00 AM with overnight fasting. The most widely used method for measurement of free testosterone in clinical laboratories is direct RIA. In general, this assay uses a 125I-labeled testosterone analog that has very low affinity for SHBG and albumin and competes with free testosterone for binding sites on an immobilized specific testosterone antibody. Although this approach provides a simple and rapid test for quantifying free testosterone, it has been pointed out that the assay method has several deficiencies; these include low antibody affinity, major biasing effects resulting from dilution of serum samples, significant binding of the analog to serum proteins, and lack of parallelism between measurements of serially diluted serum samples and free testosterone. For these reasons, the reliability of the assay that utilizes the analog-based free testosterone RIA kit has been questioned. [11–13] Other relevant evaluation was also done as needed.

### 2.1. Statistical Method

Descriptive statistics was analyzed with SPSS version 17.0 software. Continuous variables were presented as mean ± SD. Categorical variables were expressed as frequencies and percentages. Nominal categorical data between the groups were compared using the chi-squared test or Fisher's exact test as appropriate. *P* < 0.05 was considered as statistically significant.

## 3. Results

In our study, prevalence of symptomatic TDS was 48.18% (359/745). Maximum number of participants were in the 40–49 year group and least in more than 80 years group with a mean age of 54.48 ± 10.679 years (*R* = 40–93 years) (Table 2).

In our study, a significant correlation was found between aging and decline of the TT level (*r* = −0.153, *p* value = 0.004). Significant correlation was also found between aging and decline of the FT level (*r* = −0.121, *p*=0.002). Prevalence of TDS increases progressively with each decade of life, and it was found to be statistically significant (*p*=0.034). Most commonly reported ADAM questionnaire symptom was lack of energy followed by decreasing frequency by poor erection and loss of libido, and the least reported symptom was loss of height.

Mean TT level of our symptomatic TDS population was 3.287 ± 1.494 ng/ml (1.12–9.61). Overt TDS (TT < 2.50 ng/ml) and borderline TDS (TT = 2.50–3.46 ng/ml) were found in 34.82% (125/359) and 27.85% (100/359) subjects, respectively. Mean FT level of our symptomatic TDS population was 7.476 ± 2.902 pg/ml (2.18–21.76). Prevalence of biochemically confirmed TDS among symptomatic TDS population was 60.17% on the basis of FT levels.

**Figure 1:**
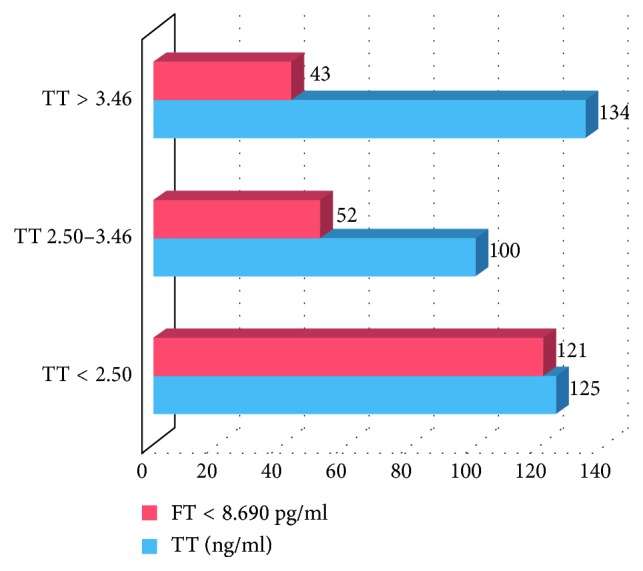
Graph showing association between TT and FT levels in the study population.

In our study, association between TT and FT levels was found to be statistically significant (*p* value < 0.001) (Figure 1). 96.8% participants with overt TDS (total T < 2.50 ng/ml) were found to have the FT level <8.690 pg/ml. The prevalence of symptomatic + biochemical TDS in our study population was 28.99% (216/745) on the basis of FT levels.

Mean body mass index (BMI) of symptomatic TDS population was 24.82 ± 2.59 (18.29–32.59). Obese (BMI > 30) and overweight (BMI = 25–30) patients made up 4.45% and 37.6% (135/359) of study population. Increasing BMI has a significant correlation with TDS (*r* = −0.156, *p*=0.003).

**Figure 2:**
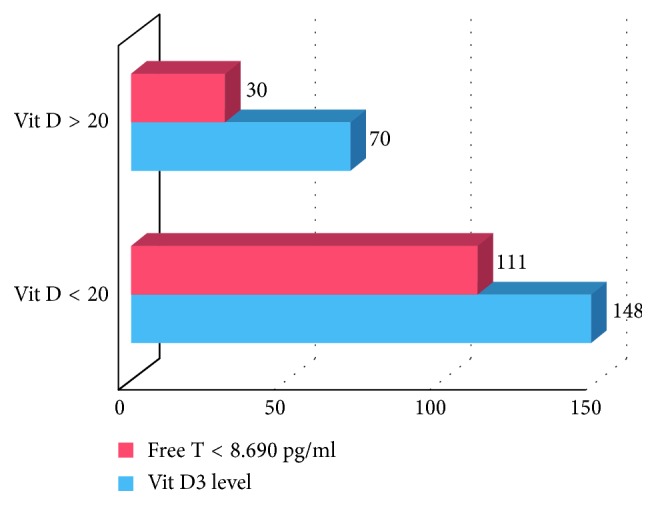
Division of study population in two groups based on vitamin D_3_ levels (*n* = 218) and prevalence of FT levels in the two subgroups (FT = free testosterone).

Prevalence of TDS in diabetic population was significantly higher as compared to nondiabetics (71.03% vs 52.8%, *p*=0.001). Similarly, significantly higher number of hypertensive patients were found to have TDS as compared to normotensives (72.89% vs 54.86%, *p*=0.001). In our study, 11.42% (51/359) of symptomatic TDS were to have metabolic syndrome (MetS). 80.39% (41/51) of participants with MetS were found to have TDS with a statistically significant association (*p*=0.003). 84.30% (27/32) participants with coronary artery disease (CAD) were found to have TDS, suggesting that patients having TDS should be evaluated for CAD/MetS, and vice versa (*p*=0.003). In our study, a significant association was found between vitamin D_3_ deficiency and TDS (*p* < 0.001), as shown in Figure 2.

## 4. Discussion

In our study, the ADAM questionnaire was administered among 745 males having aged ≥40 year. Prevalence of symptomatic TDS among study population was found to be 48.18% (359/745). Overt TDS (TT < 2.50 ng/ml) was present in 34.82% (125/359) subjects. Prevalence of biochemically confirmed TDS among symptomatic TDS population was 60.17%. Prevalence of symptomatic + biochemically confirmed TDS in study population was 28.99% (216/745). A preliminary cross-sectional study conducted by Goel et al. on 157 healthy volunteers found that the prevalence of symptomatic LOH was (67.5%) on the ADAM questionnaire [9]. Another Indian study conducted by Ashat et al. reported 40% prevalence of symptomatic andropause among men with age of 40 or above [10]. Goel et al. reported prevalence of biochemical TDS among symptomatic TDS population of male workers between ages 40 and 60 as 38.67%, and prevalence of overt TDS was 30.18% [9]. Mulligan et al. in the HIM study reported prevalence of hypogonadism to be 36.3% among men >45 years attending general clinical practice [10]. In the current study, mean serum TT levels of symptomatic TDS population was 3.287 ng/ml (SD = 1.494, range = 1.12–9.61). Mean TT level was reported to be 1.5 ng/ml by Goel et al. [9] and 3.46 ng/ml by Mulligan et al. [11].

In the current study, maximum number of symptomatic TDS participants was in the 40–49 year group and least in the >80 year group with a mean age of 54.48 ± 10.679 years (40–93 years). Similar age profile for symptoms of TDS was reported by Ashat et al. [10]. This reflects the general assumption in our Indian society that symptoms of TDS are general phenomenon of aging. Most commonly reported ADAM questionnaire symptom was lack of energy followed by decreasing frequency by poor erection and loss of libido, and the least reported symptom was loss of height. Kelleher et al. in their study found that most reported symptoms of androgen deficiency were lack of energy and reduced libido [12].

Significant correlation was also found between aging and decline of the FT level (*r* = −0.121, *p*=0.002). Prevalence of TDS increases progressively with each decade of life, and it was found to be statistically significant (*p*=0.034). Similar findings were reported in the HIM study [11]. In the current study, mean BMI of symptomatic TDS population was 24.82 ± 2.59 (18.29–32.59). In a cross-sectional pilot study, mean BMI was 22.9 in patients with TDS [9]. Increasing BMI has a significant correlation with TDS (*r* = −0.156, *p*=0.003). Similar finding was reported in the HIM study [11]. The odds ratio (2.38) for having hypogonadism was significantly higher in men with obesity, as calculated in the HIM study [11].

In the current study, prevalence of TDS in diabetic population was significantly higher as compared to nondiabetics (71.03% vs 52.8%, *p*=0.001). Khan et al. also found that testosterone levels are frequently low in men with T2DM, and the majority of these men have symptoms of hypogonadism [13]. 72.89% (78/107) hypertensive patients were found to have TDS with a prevalence of 72.89% which was significantly higher than normotensive (*p*=0.001) patients. The HIM study also calculated that the odds ratio for having hypogonadism was 2.09 and 1.84, respectively, in diabetic and hypertensive patients [11].

In our study, 11.42% (51/359) of symptomatic TDS were to have MetS based on three of the five criteria (elevated WC, elevated TGs, reduced HDL-C levels, elevated BP, and elevated FBG levels) by a new joint statement from a number of professional organizations such as IDF, the National Heart, Lung, and Blood Institute (NHLBI), WHO, the International Atherosclerosis Society, and the American Heart Association (AHA) for the clinical diagnosis of MetS [14]. 80.39% (41/51) of participants with MetS were found to have TDS with a statistically significant association (*p*=0.003). Similar association between TDS and MetS has been found by Salam et al. [14].

In our study, 84.30% (27/32) of participants with CAD were found to have TDS and association was found to be statistically significant (*p*=0.003). In their study, Seth M. et al. found that the mean testosterone levels (241–827 ng/dl) were significantly lower in CAD patients than the controls (*p* < 0.05) [15]. In our study, a significant association was found between vitamin D_3_ deficiency and TDS (*p* < 0.001). Tak YJ et al. have found similar association in a cross-sectional study. In the multiple linear regression model, 25(OH)D_3_ showed positive association with TT (*β* = 0.137, *p* < 0.001) and FT (*β* = 0.103, *p*=0.008). Vitamin D_3_ deficiency (25(OH)D_3_ < 20 ng/ml) was associated with an increased risk of deficiencies of TT (<2.30 ng/ml) (odds ratio (OR): 2.65; CI: 1.21–5.78, *p*=0.014) and free testosterone (<6.50 pg·ml^−1^) (OR: 1.44; 95% CI: 1.01–2.06, *p*=0.048) [16]. Patients with hypogonadism have frequently low levels of 25-hydroxyvitamin D due to impairment of the hydroxylating enzyme CYP2R1 in the testis [17].

## 5. Conclusion

Testosterone deficiency syndrome (TDS) is a clinical and biochemical syndrome associated with aging and characterized by symptoms affecting multiple organ systems. The prevalence of symptomatic TDS and biochemically confirmed TDS is 60.17% and 28.99%, respectively. Its prevalence increases progressively with each decade of life. Prevalence was higher in patients with diabetes, hypertension, obesity, metabolic syndrome, and coronary artery disease. A significant association was found between vitamin D_3_ deficiency and TDS.

## Figures and Tables

**Table 1:** Saint Louis University androgen deficiency in aging male (ADAM) questionnaire.

1	Do you have a decrease in libido or sex drive?	Yes/no
2	Do you have a lack of energy?	Yes/no
3	Do you have a decrease in strength and/or endurance?	Yes/no
4	Have you lost weight?	Yes/no
5	Have you noticed a decreased “enjoyment of life”?	Yes/no
6	Are you sad and/or grumpy?	Yes/no
7	Are your erections less strong?	Yes/no
8	Have you noticed a recent deterioration in your ability to play sports?	Yes/no
9	Are you falling asleep after dinner?	Yes/no
10	Has there been a recent deterioration in your work performance?	Yes/no

**Table 2:** Prevalence of symptomatic and biochemically confirmed TDS in different age groups.

Age group	ADAM +ve	Biochemically +ve	Prevalence (%)
40–49 years	136	73	53
50–59 years	121	75	61.98
60–69 years	67	44	65
70–79 years	25	17	68
>80 years	10	7	70

## Data Availability

The data used to support the findings of this study are available from the corresponding author upon request.

## References

[1] Feldman H. A., Longcope C., Derby C. A. (2002). Age trends in the level of serum testosterone and other hormones in middle-aged men: longitudinal results from the Massachusetts male aging study. *Journal of Clinical Endocrinology & Metabolism*.

[2] Harman S. M., Metter E. J., Tobin J. D., Pearson J., Blackman M. R. (2001). Longitudinal effects of aging on serum total and free testosterone levels in healthy men. *Journal of Clinical Endocrinology & Metabolism*.

[3] Kaufman J. M., Vermeulen A. (1997). Declining gonadal function in elderly men. *Baillière’s Clinical Endocrinology and Metabolism*.

[4] Zmuda J. M., Cauley J. A., Kriska A., Glynn N. W., Gutai J. P., Kuller L. H. (1997). Longitudinal relation between endogenous testosterone and cardiovascular disease risk factors in middle-aged men: a 13-year follow-up of former multiple risk factor intervention trial participants. *American Journal of Epidemiology*.

[5] Morales A., Wein A. J. (2012). Androgen deficiency in the aging male. *Campbell-Walsh Urology*.

[6] Wang C., Nieschlag E., Swerdloff R. (2008). Investigation, treatment and monitoring of late-onset hypogonadism in males: ISA, ISSAM, EAU, EAA and ASA recommendations. *European Journal of Endocrinology*.

[7] Nieschlag E., Swerdloff R., Behre H. M. (2005). Investigation, treatment and monitoring of late-onset hypogonadism in males. ISA, ISSAM, and EAU recommendations. *European Urology*.

[8] Lunenfeld B., Mskhalaya G., Zitzmann M. (2015 Mar). Recommendations on the diagnosis, treatment and monitoring of hypogonadism in men hypogonadism in men. *The Aging Male*.

[9] Goel A., Sinha R. J., Dalela D., Sankhwar S., Singh V. (2009). Andropause in Indian men: a preliminary cross-sectional study. *Journal of Urology*.

[10] Ashat M., Puri M., Singh A., Sarpal S. S., Goel N. K., Kaushal B. (2011). Awareness of andropause in males, a north Indian study. *Indian Journal of Medical Sciences*.

[11] Morley J. E., Perry H. M., Kevorkian R. T., Patrick P. (2006). Comparison of screening questionnaires for the diagnosis of hypogonadism. *Maturitas*.

[12] Kelleher S., Conway A. J., Handelsman D. J. (2004). Blood testosterone threshold for androgen deficiency symptoms. *Journal of Clinical Endocrinology & Metabolism*.

[13] Khan I., Kant C., Samaria A. (2012). Assessment of hypogonadism with reference to clinical features and serum testosterone levels in Asian-Indian male type 2 diabetics. *Indian Journal of Clinical Practice*.

[14] Salam R., Kshetrimayum A. S., Keisam R. (2012). Testosterone and metabolic syndrome: the link. *Indian Journal of Endocrinology and Metabolism*.

[15] Seth M., Sachdeva A., Saharoy P., Seth S., Madaan H. (2011). Relationship of testosterone levels in males with coronary Heart DIsease. *International Journal of Pharma and Bio Sciences*.

[16] Tak Y. J., Lee J. G., Kim Y. J. (2015). Serum 25-hydroxyvitamin D levels and testosterone deficiency in middle-aged Korean men: a cross-sectional study. *Asian Journal of Andrology*.

[17] Foresta C., Calogero A., Lombardo F., Lenzi A., Ferlin A. (2015). Late-onset hypogonadism: beyond testosterone. *Asian Journal of Andrology*.

